# Prescriptions and Dispensing

**Published:** 1907-10-26

**Authors:** 


### THERAPEUTICS.

### PRESCRIPTIONS AND DISPENSING.

(*Continued from page 67.*)

### III. MEDICINES IN LIQUID FORM.

Many of the commoner soluble salts are sometimes kept in solution, and in that case instead of weighing out the amount of solid required, the corresponding volume of solution is measured. There is no objection to this plan, provided it is kept within proper limits; thus only those salts should be so dealt with that are quite stable in solution, salts of organic acids being generally unsuitable on account of the tendency of their solutions to develop growths of a fungous nature, while some salts, such as the official ammonium carbonate, undergo gradual decomposition in water. The usefulness of aqueous solutions is further limited by the fact that they can only be employed in a prescription in which the desired vehicle is water; they are not applicable in those cases in which a medicated water or an infusion is the vehicle, unless concentrated infusions are employed, a point we shall deal with shortly.

It is, of course, essential, if stock solutions are used, that they should be accurately prepared, and it is necessary to be on guard against errors arising from confusion between grain-measure and minim-measure. A solution of potassium bromide, for example, may be conveniently made to contain 60 grains in 4 fluid drachms; but this should not be spoken of as a 1 in 4 solution, since a fluid drachm contains 60 minims but only 54.7 grain-measures.

The solubilities of salts are not familiar to all prescribers, and this often leads to difficulties in dispensing; a larger quantity may be prescribed than will dissolve in the amount of vehicle. The degree of solubility of a solid in a liquid is, of course, largely affected by temperature, and as a rule the solubility is greater at a higher temperature than at a lower. This is not a universal law, phosphates being a well-known example to the contrary; but it is true of most salts. It might be suggested, therefore, that the excess of the salt should be brought into solution by heating the vehicle, or heating the two together until the solution is complete. Thus, in the following lotion—

| | | |
|---|---|---|
| ℞ | Acidi Borici ... ... ... ... ... | ʒiij. |
| | Sodii Chloridi ... ... ... ... ... | ʒij. |
| | Aquam Rosæ ad ... ... ... ... ... | ℥vj. |

the boric acid all dissolves if the mixture be warmed to about 45°C. When the liquid has become cold again, however, the excess of boric acid is no longer held in solution, but crystallises out. This may not occur for some time, as a solution will often remain supersaturated when undisturbed; but if crystallisation takes place after the medicine is in the patient's hands the dissatisfaction of the latter will be just as great as if it had been received in that condition. The prescriber must not only consider changes that may occur in the course of compounding, but he must also accustom himself to look ahead and foresee reactions that may only take place after an interval, and provide properly against them. The above lotion may be intended for use hot, in which case the patient should be warned that there will be a white precipitate, which will disappear when the contents of the bottle are heated; if, on the other hand, the lotion is for use cold, the prescription is not a good one, containing as it does too much boric acid.

There are other effects of heat which must be taken into consideration. In the following prescription—

| | | |
|---|---|---|
| ℞ | Sodii Bicarbonatis ... ... ... ... | ʒvj. |
| | Tincturæ Gentianæ compositæ ... ... | ʒij. |
| | Syrupi ... ... ... ... ... ... | ℥ss. |
| | Aquam ad ... ... ... ... ... ... | ℥vj. |

the sodium bicarbonate will not all dissolve in $5\frac{1}{4}$ ounces of cold water; but if it is boiled with the water for a few minutes it not only dissolves, but none of it is deposited on cooling. Such a use of heat, however, is no more permissible here than it was in the former case; solution of a bicarbonate is decomposed by boiling, carbon dioxide escaping, and the corresponding carbonate remaining in the liquid. The excess of bicarbonate is not deposited because the salt has been changed to carbonate, and if the prescriber desired this he would order carbonate, and not bicarbonate, in the first instance.

The application of heat is necessary in preparing some other forms of medicine, but in the dispensing of mixtures the cases are extremely rare in which it should be used. The general rules must, of course, be that heat is not to be used to produce a change, such as increased solubility, which will be reversed again on cooling; and, on the other hand, it must not be employed to produce a permanent change, where the result of this change is the administration to the patient of a different chemical substance from that ordered. If it is desirable to save time by using hot water to dissolve a slowly soluble salt, or to employ heat in any other way, one must be satisfied that the case is one outside these two general rules.

The following mixture presents an instance of what may occur in such cases as those we have been discussing:—

| | | |
|---|---|---|
| ℞ | Potasii Chloratis ... ... ... ... | ℥ss. |
| | Syrupi Zingiberis ... ... ... ... | ʒvj. |
| | Infunsum Aurantii ad ... ... ... | ℥vj. |

The quantity of potassium chlorate is more than the vehicle will dissolve at the ordinary room temperature, and the excess must remain as a sediment of fine powder. If the patient happens to keep a part of the mixture for some time the powdered sediment is likely to be gradually replaced by crystals, which can no longer be evenly distributed by shaking or mixing, and a complaint to the doctor is sure to ensue. The explanation of the change is that the medicine has been exposed to variations of temperature, either large or small; when the temperature rises a little more chlorate goes into solution, and when it falls this excess is deposited, but now in crystals; at the next rise of temperature more of the powder, not of the newly formed and less soluble crystals, will dissolve, to be in turn deposited as crystals, and by the continuance of this alternating process the powder gradually comes to be all replaced by crystals.

